# *NN-align*. An artificial neural network-based alignment algorithm for MHC class II peptide binding prediction

**DOI:** 10.1186/1471-2105-10-296

**Published:** 2009-09-18

**Authors:** Morten Nielsen, Ole Lund

**Affiliations:** 1 Center for Biological Sequence Analysis, Department of Systems Biology, Technical University of Denmark, Building 208, DK-2800 Lyngby, Denmark

## Abstract

**Background:**

The major histocompatibility complex (MHC) molecule plays a central role in controlling the adaptive immune response to infections. MHC class I molecules present peptides derived from intracellular proteins to cytotoxic T cells, whereas MHC class II molecules stimulate cellular and humoral immunity through presentation of extracellularly derived peptides to helper T cells. Identification of which peptides will bind a given MHC molecule is thus of great importance for the understanding of host-pathogen interactions, and large efforts have been placed in developing algorithms capable of predicting this binding event.

**Results:**

Here, we present a novel artificial neural network-based method, *NN-align that *allows for simultaneous identification of the MHC class II binding core and binding affinity. *NN-align *is trained using a novel training algorithm that allows for correction of bias in the training data due to redundant binding core representation. Incorporation of information about the residues flanking the peptide-binding core is shown to significantly improve the prediction accuracy. The method is evaluated on a large-scale benchmark consisting of six independent data sets covering 14 human MHC class II alleles, and is demonstrated to outperform other state-of-the-art MHC class II prediction methods.

**Conclusion:**

The *NN-align *method is competitive with the state-of-the-art MHC class II peptide binding prediction algorithms. The method is publicly available at http://www.cbs.dtu.dk/services/NetMHCII-2.0.

## Background

Major histocompatibility complex (MHC) molecules play an essential role in host-pathogen interactions determining the onset and outcome of many host immune responses. Only a small fraction of the possible peptides that can be generated from proteins of pathogenic organisms actually generate an immune response. MHC class II molecules present peptides derived from proteins taken up from the extracellular environment. They stimulate cellular and humoral immunity against pathogenic microorganisms through the actions of helper T lymphocytes. In order for a peptide to stimulate a helper T lymphocyte response, it must bind MHC II in the endocytic organelles [1].

The MHC class I molecule is highly specific and binds a limited set of peptides of a narrow length distribution [2]. In contrast to this, the MHC class II molecule is highly promiscuous both with respect to composition and length of the peptide ligands [3,4]. During the last decade, large efforts have been invested in developing methods to allow for *in silico *screening of pathogenic organisms with the purpose of identifying peptides that will bind MHC class II molecules in a given host [5-20]. The majority of these methods are trained on limited data sets covering a single or a few MHC molecules. The binding of a peptide to a given MHC molecule is predominantly determined by the amino acids present in the peptide-binding core. However, peptide residues flanking the binding core (so-called peptide flanking residues, PFR) do also to some degree affect the binding affinity of a peptide [21,22]. Most published methods for MHC class II binding prediction however focus on identifying the peptide-binding core only, ignoring the effects on the binding affinity of PFRs.

In two recent publications, we have demonstrated i) how a stabilization matrix method (*SMM-align*) could be applied to simultaneously identify the peptide-binding core and predict the binding strength [20], and ii) how this peptide core alignment together with information about the peptide flanking residues could be integrated in a neural network-based algorithm that allows for pan-specific HLA-DR binding prediction (*NetMHCIIpan*) [23]. Both the *SMM-align *and the *NetMHCIIpan *methods have in recent benchmarks been shown to be among the best publicly available methods for HLA-DR peptide binding prediction [18,24].

Here, we show how an artificial neural network-based alignment method, *NN-align*, can significantly outperform both the *SMM-align *and *NetMHCIIpan *methods. The *NN-align *method includes explicit encoding of the peptide flanking residues in terms of amino acid composition and length, as well as a novel scheme for neural network training that deals with the data redundancy inherent in the peptide data due to multiple examples of identical binding cores. The *NN-align *method is trained on a large data set of more than 14,000 quantitative peptide MHC binding values covering 14 HLA-DR alleles. The performance is evaluated on five independent data sets and its performance is compared to the best publicly available state-of-the-art MHC class II prediction methods.

## Methods

### Data

A quantitative IEDB HLA-DR restricted peptide-binding data set was obtained from the data published by Nielsen et al. [23]. The data set comprises 14 HLA-DR alleles each characterized by at least 420 and up to 5166 peptide binding data points. To minimize the peptide overlap between training and testing data, the binding data for each HLA-DR allele was partitioned into 5 data sets using the approach outlined by Nielsen et al. [20] minimizing the sequence overlap between the training and test data. Each data set and corresponding partition is made available online at http://www.cbs.dtu.dk/suppl/immunology/NetMHCII-2.0.php.

The method was evaluated further on five independent data sets taken from recent MHC class II benchmark publications. The HLA-DR class II ligand data set from the SYFPEITHI database [4] was taken from Nielsen et al. [23]. Only ligands restricted by one of the 14 HLA-DR alleles in the quantitative binding data set were included. This SYFPEITHI data set consists of 475 MHC ligands. The peptide core data set was also taken from Nielsen et al. [23] to evaluate the ability of the proposed method to identify the core of HLA-DR binding peptides. As a further evaluation, the method was evaluated against the benchmark data sets published by Lin et al. [24] and Wang et al. [18]. The Lin data set data set contains binding affinities of 103 overlapping peptides to seven common HLA-DR molecules (DRB1*0101, 0301, 0401, 0701, 1101, 1301, and 1501). Only six of the seven alleles were included since the DRB1*1301 allele is not covered by the data in the quantitative training data set. All methods from the Lin benchmark that cover all six alleles and which were labeled as "best performing" by Lin at al. were included in the benchmark. The Wang et al. benchmark consists of quantitative binding data to 14 HLA-DR alleles. In this benchmark, the predictive performance of the NN-based method was evaluated using 10-fold cross-validation. Finally, to investigate how the performance of the NN-based method depends on the inherent similarity in the peptide data, the method was benchmarked using the El-Manzalawy benchmark data set [25]. Three data sets with different degrees of inherent peptide similarity were selected from the IEDB benchmark data set: UPDS; unique peptides from the IEDB data base, SRDS1; sequence similarity reduced UPDS data excluding peptides sharing 9 mer subsequences, and SRDS2; sequence similarity reduced SRDS1 data ensuring maximum 80% similarity between pairs of peptides. Three support vector machine (SVM) based MHC class II binding prediction methods were included in the El-Manzalawy et al. benchmark; composition transition distribution (CTD), local alignment kernel (LA), and k-spectrum kernel (5-spectrum). The performance of the different methods was evaluated using the AUC measure since this is the only measure provided in the El-Manzalawy paper that does not depend on binding affinity classification.

### Method

*NN-align *was implemented as a conventional feed-forward artificial neural network method [26]. The method consists of a two-step procedure that simultaneously estimates the optimal peptide binding register (core) and network weight configuration. Initially, all network weights were assigned random values. Given this set of network weights, the core of a given peptide was identified as the highest scoring of all 9 mers contained within the peptide. The score of a 9 mer peptide was calculated using the conventional feed-forward algorithm. The network weights were updated using gradient descent back-propagation. Given a peptide core alignment, the weights were updated to lower the sum of squared errors between the predicted binding score and the measured binding affinity target value. The quantitative binding data contains a large degree of redundancy since many peptide data points have been measured repeatedly with single amino acid mutations in order to identify for instance the effect of the amino acids flanking the peptide-binding core. To limit the effect of such data redundancy, the network back-propagation was modified so that the step-size of back-propagation was divided by the binding core redundancy of the given peptide. The binding core redundancy was calculated using a Hobohm-1 algorithm [27] to define clusters of identical binding cores. For a peptide belonging to a cluster containing five peptides with identical binding core, the step-size of back-propagation was thus divided by five. The identification of peptide clusters was done repeatedly during the network training based on the given binding core configuration.

The 9 mer peptide was encoded to the network as described for the *NetMHCIIpan *by Nielsen et al. [23]. In brief, the peptide core was presented to the network using Blosum encoding, where each amino acid was encoded by the BLOSUM log-odds vector [28,29]. The peptide flanking regions (PFR) were presented as the average BLOSUM substitution frequency vector over a maximum length of three amino acids. The PFR length was encoded as L_PFR_/3, 1-L_PFR_/3, where L_PFR _is the length of the PFR (between 0 and 3), and the peptide length was encode as L_PEP_, 1-L_PEP_, where L_PEP _= 1/(1+exp((L-15)/2)) and L is the peptide length. To impose an amino acid preference at the P1 position of the binding core, a log-odds position specific scoring matrix (PSSM) was constructed using the Gibbs sampler method for class II binding prediction [10] using the peptide binders in the training data for the allele in question. Next, the core P1 amino acid was encoded as the P1-PSSM score for that given amino acid, i.e. for a given peptide binding register, one additional value was added to the network input as the score of the core P1 amino acid at the P1 position in the PSSM. For each peptide core, the input to the neural network thus consisted of the peptide sequence (9×20 = 180 inputs), the PFRs (2×20 = 40 inputs), the peptide length (2 inputs), the length of the C and N terminal PFR's (2×2 = 4 inputs), and the P1-PSSM score (1 input) resulting in a total of 227 input values. The peptide binding affinity IC50 values were encoded to the neural network as log-transformed values, using the relation 1-log (aff)/log(50000), where aff is the measured binding affinity (IC50) in nM units [29].

The networks were trained using cross-validation. Network ensembles were trained with 2, 10, 20, 40 and 60 hidden neurons, respectively. The procedure of i) identifying the optimal peptide core, and of ii) updating the network weights to lower the predictive error was repeated for 500 cycles for each network architecture. Since the "search landscape" has a large set of local minima, each with close to identical performance values, the network training was run 10 times, each time with different initial configuration values, for each network architecture. This led to a significantly improved prediction accuracy (data not shown). In total 50 (5 architectures*10 seeds) networks were created for each training/test set configuration. For each training/test set configuration, the 10 networks with the highest test-set Pearson correlation coefficient were selected to form the final network ensemble. The binding core of a given peptide was assigned by a majority vote of the networks in the ensemble.

### Statistical tests

All statistical comparisons were made using one-tailed binomial tests. For each comparison, it is calculated how often one method outperforms the other (excluding ties), and based on these numbers one-tailed p-values were calculated. P-values less than 0.05 were taken to be significant.

### SMM-align and NetMHCIIpan methods

The *SMM-align *method was trained as described by Nielsen et al. [20] on the IEDB quantitative data set combined with HLA-DR ligand data obtained from the SYFPEITHI database [30]. The *NetMHCIIpan *method was used via the online server-link http://www.cbs.dtu.dk/services/NetMHCIIpan.

## Results

### The quantitative IEDB benchmark data set

The predictive performance of the artificial neural network (NN) based methods on the quantitative IEDB benchmark data set is shown in Table 1. The performance was estimated using five-fold cross-validation. In each cross-validation, 1/5 of the data were left out for evaluation and the remaining 4/5 were used for a four-fold cross-validated training as described in the methods section. The four-fold cross-validated training resulted in an ensemble of 40 networks. The predictive binding affinity for the peptides in the evaluation set was next calculated as a simple average over the 40 network predictions, and the peptide-binding core was identified by a majority vote. In this approach, no peptide from the evaluation set was included in either the network training or the identification of the optimal network ensemble.

**Table 1:** Predictive performance for the 14 HLA-DR alleles in the quantitative IEDB benchmark dataset.

Allele	#	*TEPITOPE*	*SMM-align*	NN	NN-W	NN-P1	NN-W-P1	NN-xPFR
DRB1*0101	5166	0.720	0.802	**0.837**	0.836	0.833	0.833	0.813
DRB1*0301	1020	0.664	0.795	0.808	0.816	**0.817**	**0.817**	0.778
DRB1*0401	1024	0.716	0.751	**0.768**	0.771	0.764	0.766	0.764
DRB1*0404	663	0.770	0.801	0.815	**0.818**	0.816	0.817	0.814
DRB1*0405	630	0.759	0.789	0.771	0.781	0.779	**0.784**	0.773
DRB1*0701	853	0.761	0.812	**0.844**	0.841	0.839	0.838	0.813
DRB1*0802	420	0.766	0.787	0.826	**0.832**	0.820	0.822	0.802
DRB1*0901	530		0.655	0.623	0.616	0.618	**0.640**	0.623
DRB1*1101	950	0.721	0.796	0.822	**0.823**	0.818	0.813	0.802
DRB1*1302	498	0.652	0.785	0.822	**0.831**	0.822	0.824	0.811
DRB1*1501	934	0.686	0.727	0.754	0.758	0.754	0.754	**0.761**
DRB3*0101	549		0.836	**0.855**	0.844	0.838	0.841	0.831
DRB4*0101	446		0.793	0.811	0.811	0.815	**0.818**	0.813
DRB5*0101	924	0.680	0.761	0.789	**0.797**	0.790	0.790	0.787

Ave		0.718	0.778	0.796	0.798	0.795	0.797	0.785
Ave*		0.716	0.785	0.809	0.810	0.807	0.808	0.793

The performance was estimated using five-fold cross-validation and is given as the area under the ROC curve (AUC) calculated using a binding affinity threshold of 500 nM. *SMM-align *is the *SMM-align *method described by Nielsen et al. [20] re-trained on the quantitative benchmark data set. *TEPITOPE *refers to the method developed by Sturniolo et al. [17]. *NN *is the standard NN-based method, *NN-W *is the NN-based method including data redundancy step-size rescaling, *NN-P1 *is the NN-based method including PSSM-P1 amino acid encoding, *NN-W-P1 *is the NN-based method including data redundancy step-size rescaling and PSSM-P1 amino acid encoding. *NN-xPFR *is the *NN-W-P1 *method excluding peptide-flanking residue encoding. For each allele, the best performing NN method is highlighted in bold and the best performing of all methods is underlined. Ave is the average per-allele performance over all 14 alleles, and Ave* is the average predictive performance over the 14 alleles weighted by the number of data points for each allele.

Table 1 shows the predictive performance in terms of AUC [31] (area under the receiver operator curve) of the different NN-based methods. Performance values in terms of the Pearson's correlation coefficient are shown in supplementary material [see Additional file 1]. Further, the predictive performance of the *SMM-align *[20] and *TEPITOPE *[17] methods is given. The *TEPITOPE *method does not produce prediction values that are linearly related to the log-transformed binding affinities, which is why for this method only AUC values are reported. The performance is shown for five different versions of the NN-based method, each including different degrees of sophistication in the training algorithm. *NN *is the standard NN-based method, *NN-W *is the NN-based method including data redundancy step-size rescaling, *NN-P1 *is the NN-based method including PSSM-P1 amino acid encoding, *NN-W-P1 *is the NN-based method including both data redundancy step-size rescaling and PSSM-P1 amino acid encoding, and *NN-xPFR *is the *NN-W-P1 *method excluding peptide-flanking residue encoding.

From Table 1, it is clear that the NN-based methods that include data redundancy step-size rescaling (*NN-W *and *NN-W-P1*) significantly outperformed (p < 0.05) their non-rescaled counterparts (*NN *and *NN-P1*). When compared to the *SMM-align *and *TEPITOPE *methods, the NN-based methods all showed a significantly improved predictive performance (p < 0.05, in all cases). Only for two alleles (DRB1*0405 and DRB1*0901) did the *SMM-align *method outperform NN-based methods, while TEPITOPE did not perform best for any of the alleles.

The per-allele binomial statistical test applied here places equal weight on all alleles and does not take into account the large differences in the peptide data available for each allele. To place a relative weight on the performance of the different alleles, Table 1 includes a performance value for each method calculated as the mean weighted by the number of peptides per allele. However, a pooled statistical test on all peptide data would favor methods being able to accurately predict binding for the alleles described by most data over other methods. In the per-allele binomial test for instance, the methods *NN *and *NN-P1 *have comparable performance. *NN *has the best performance for 7 alleles and the *NN-P1 *method performs best for 5 (see Table 1). This difference is not significant. When performing a binomial test on the predicted error per peptide for the pooled set of 14,607 peptides, the difference between the two methods comes out highly significant in favor of the *NN *method (p < 0.007). However, 35% of the peptide binding data are measured for the DRB1*0101 allele. The *NN *method performs best of the four NN-based methods on the HLA-DRB1*0101 allele and pooling the data hence favors this method over the other. When performing the same test on the pooled peptide data excluding the DRB1*0101 data, the two methods come out with comparable performance (p < 0.20). Since it is essential to demonstrate that a prediction method has high predictive performance across a large set of different alleles, we therefore here limit the statistical tests to per-allele binomial tests.

Comparing the performance of the NN-based method with (*NN-W-P1*) and without peptide-flanking residue encoding (*NN-xPFR*) demonstrated the importance of the latter for improved prediction accuracy. For 13 of the 14 alleles, did the inclusion of PFR improve the prediction accuracy (p < 0.005). The direct and linear relationship between the *NN-W-P1 *prediction score and the measured binding affinity is apparent when calculating a least square linear fit between the prediction score and the log-transformed binding affinities. Here, the average slope was 0.86 ± 0.10 and the intercept 0.05 ± 0.05 (data not shown).

The NN-based method with P1-PSSM amino acid encoding (*NN-W-P1*) showed a lower performance compared to the method without P1-PSSM amino acid encoding (*NN-W*). However, when investigating the amino acid distribution at the P1 position of the predicted binding cores of the two methods, it became apparent that the *NN-W-P1 *method produces a P1 binding core amino acid preference that is in stronger accordance with prior experimental knowledge than the *NN-W *method. Only hydrophobic amino acids have experimentally been found to be allowed at the P1 position in the binding core of HLA-DR molecules [17,32]. For the 14 alleles in Table 1, we found that the *NN-W *method on average assigned 30% of the predicted binders to have non-conventional P1 amino acids, whereas the corresponding value for the *NN-W-P1 *method was 16%.

### Identification of the peptide-binding core

To further investigate the effect of this difference in P1 binding core amino acid preference, the ability to correctly identify the binding core of HLA-DR binding peptides was investigated. The HLA-DR binding core data set was taken from Nielsen et al. [23] and consists of 15 peptides where the binding register to a HLA-DR allele has been determined from the structure of the protein complex. The result of the benchmark is shown in Table 2. From this table, it is clear that the *NN-W-P1 *method was to a higher degree capable of identifying peptide-binding cores than *NN-W*. *TEPITOPE *correctly identified all 15 binding cores, the *NN-W-P1 *method (together with *SMM-align *and *NetMHCIIpan*) misaligned one peptide by a single amino acid residue, whereas the *NN-W *method misaligned 5 peptides.

**Table 2:** Identification of peptide binding cores.

Allele	Peptide	Core	*TEPITOPE*	*SMM-align*	*NetMHCIIPan*	*NN-W-P1*	*NN-W*
DRB1*0101	**AGFKGEQGPKGEPG**	**FKGEQGPKG**	**FKGEQGPKG**	**FKGEQGPKG**	**FKGEQGPKG**	**FKGEQGPKG**	**FKGEQGPKG**
DRB1*0101	**GELIGILNAAKVPAD**	**IGILNAAKV**	**IGILNAAKV**	**IGILNAAKV**	**IGILNAAKV**	**IGILNAAKV**	**IGILNAAKV**
DRB1*0101	**PEVIPMFSALSEGATP**	**VIPMFSALS**	**VIPMFSALS**	**VIPMFSALS**	**VIPMFSALS**	**VIPMFSALS**	**VIPMFSALS**
DRB1*0101	**PKYVKQNTLKLAT**	**YVKQNTLKL**	**YVKQNTLKL**	**YVKQNTLKL**	**YVKQNTLKL**	**YVKQNTLKL**	**YVKQNTLKL**
DRB1*0101	**VGSDWRFLRGYHQYA**	**WRFLRGYHQ**	**WRFLRGYHQ**	**WRFLRGYHQ**	**WRFLRGYHQ**	**WRFLRGYHQ**	**WRFLRGYHQ**
DRB1*0101	**AFVKQNAAALA**	**FVKQNAAAL**	**FVKQNAAAL**	**VKQNAAALA**	**FVKQNAAAL**	**FVKQNAAAL**	**FVKQNAAAL**
DRB1*0101	**AAYSDQATPLLLSPR**	**YSDQATPLL**	**YSDQATPLL**	**YSDQATPLL**	**YSDQATPLL**	**YSDQATPLL**	**YSDQATPLL**
DRB1*0301	**PVSKMRMATPLLMQA**	**MRMATPLLM**	**MRMATPLLM**	**MRMATPLLM**	**MRMATPLLM**	**MRMATPLLM**	**VSKMRMATP**
DRB1*0401	**AYMRADAAAGGA**	**MRADAAAGG**	**MRADAAAGG**	**MRADAAAGG**	**YMRADAAAG**	**YMRADAAAG**	**AYMRADAAA**
DRB1*0401	**PKYVKQNTLKLAT**	**YVKQNTLKL**	**YVKQNTLKL**	**YVKQNTLKL**	**YVKQNTLKL**	**YVKQNTLKL**	**KYVKQNTLK**
DRB1*1501	**ENPVVHFFKNIVTPR**	**VHFFKNIVT**	**VHFFKNIVT**	**VHFFKNIVT**	**VHFFKNIVT**	**VHFFKNIVT**	**VVHFFKNIV**
DRB1*1501	**ENPVVHFFKNIVTPRGGSGGGGG**	**VHFFKNIVT**	**VHFFKNIVT**	**VHFFKNIVT**	**VHFFKNIVT**	**VHFFKNIVT**	**VVHFFKNIV**
DRB5*0101	**GGVYHFVKKHVHES**	**YHFVKKHVH**	**YHFVKKHVH**	**YHFVKKHVH**	**YHFVKKHVH**	**YHFVKKHVH**	**YHFVKKHVH**
DRB5*0101	**NPVVHFFKNIVTPRTPPPSQ**	**FKNIVTPRT**	**FKNIVTPRT**	**FKNIVTPRT**	**FKNIVTPRT**	**FKNIVTPRT**	**FKNIVTPRT**
DRB5*0101	**VHFFKNIVTPRTPGG**	**FKNIVTPRT**	**FKNIVTPRT**	**FKNIVTPRT**	**FKNIVTPRT**	**FKNIVTPRT**	**FKNIVTPRT**

Correct			15/15	14/15	14/15	14/15	10/15

15 HLA-DR restricted peptides compiled from the protein database [32]. The columns in the table state the HLA restriction, the peptide sequence, the binding core as determined from the protein structure, followed by the binding core as predicted by *TEPITOPE, SMM-align, NetMHCIIpan*, *NN-W-P1 *(the NN-based method including data redundancy step-size rescaling and P1-PSSM encoding), and *NN-W *(the NN-based method including data redundancy step-size rescaling), respectively. Erroneous predictions are underlined.

### Identifying endogenously presented peptides

We next turned to the benchmark of HLA-DR endogenously presented ligands. The benchmark was performed as described by Nielsen et al [23]. In short, the ligand source protein was split into overlapping peptide sequences of the length of the ligand. All peptides except the annotated HLA ligand were taken as negatives. This is a very strong assumption since suboptimal peptides that could be presented on the HLA molecule are counted as negatives. This setup thus most likely underestimated the predictive performance, but the effect should be equal for all methods. For each protein-HLA ligand pair, the predictive performance was estimated as the AUC value. In the benchmark, we compared the predictive performance of the NN-based method including data redundancy step-size rescaling with (*NN-W-P1*) and without (*NN-W*) PSSM-P1 amino acid encoding to that of *TEPITOPE *and *NetMHCIIpan *[23]. The *SMM-align *method was not included here since it was trained on both quantitative and HLA ligand binding data [20]. The summary of this benchmark is shown in Table 3.

**Table 3:** The HLA-DR ligand benchmark.

Allele	N	*NetMHCIIpan*	*TEPITOPE*	*NN-W-P1*	*NN-W*	*NN-xPFR*
DRB1*0101	37	0.873	0.883	**0.899**	0.882	0.863
DRB1*0301	26	0.882	0.837	0.862	**0.906**	0.788
DRB1*0401	209	0.865	0.876	**0.865**	0.843	0.848
DRB1*0404	46	0.817	0.790	**0.776**	0.772	0.770
DRB1*0405	35	0.848	0.809	**0.892**	0.866	0.878
DRB1*0701	36	0.687	0.711	**0.761**	0.754	0.757
DRB1*0802	1	0.982	0.914	**0.984**	0.979	0.959
DRB1*0901	4	0.865		0.867	0.864	**0.878**
DRB1*1101	27	0.873	0.863	**0.894**	0.876	0.881
DRB1*1302	21	0.605	0.761	**0.702**	0.687	0.681
DRB1*1501	12	0.770	0.729	0.767	0.766	**0.776**
DRB3*0101	2	0.957		0.680	**0.730**	0.681
DRB4*0101	4	0.471		**0.540**	0.492	0.496
DRB5*0101	15	0.840	0.853	**0.877**	0.819	0.851

**Ave**		0.810		0.812	0.804	0.794
Ave*		0.830		0.842	0.824	0.824
Ave**		0.822	0.821	0.844	0.834	0.824

The benchmark data set consists of 475 HLA-DR restricted ligands downloaded from the SYFPEITHI database of MHC ligands covering 14 HLA-DR alleles. The predictive performance was estimated in terms of the AUC as described in the text. Ave is the average per-allele performance over all 14 alleles. Ave* is the average predictive performance over all 475 ligand/HLA-DR pairs. Ave** is the average per allele performance over the 11 alleles covered by the *TEPITOPE *method. *NetMHCIIpan *is the HLA-DR pan-specific method described by Nielsen et al. [23]. *TEPITOPE *refers to the method developed by Sturniolo et al [17]. *NN-W-P1 *is the NN-based method including data redundancy step-size rescaling and PSSM-P1 amino acid encoding. *NN-W *is the NN-based method including data redundancy step-size rescaling. *NN-xPFR *is the *NN-W-P1 *method excluding peptide flanking residue encoding. For each allele, the best performing NN method is highlighted in bold and the best performing of all methods is underlined.

For the HLA-DR ligand benchmark, the *NN-W-P1 *method including P1-PSSM encoding clearly outperforms its counterpart, *NN-W*, not including P1-PSSM encoding. Only for two alleles did *NN-W *outperform the *NN-W-P1 *method making this difference highly statistically significant (p < 0.01). Furthermore, the *NN-W-P1 *method significantly outperformed both the *NetMHCIIpan *(p < 0.05) and *TEPITOPE *(p < 0.05) methods. Likewise, it is clear that the inclusion of peptide-flanking residues significantly improved the predictive performance of the *NN-W-P1 *method (p < 0.002).

### The Wang el al. benchmark data set

The evaluation of the NN-based method on the Wang et al. benchmark is shown in Table 4. From this benchmark, it is clear that the *NN-W-P1 *and *NN-W *methods have similar predictive performance and that they significantly outperformed all other methods in the benchmark (p < 0.01 in all cases). The NN-based methods were outperformed only for one allele (DRB1*0801).

**Table 4:** Predictive performance in terms of the AUC on the Wang benchmark data set.

Allele	*ARB*	*MHC2Pred*	*MHCpred*	*Propred*	*Rankpep*	*SMM-align*	*SVRMHC*	*SYF*	*Cons*	*NN-W-P1*	*NN-W*
DRB1*0101	0.76	0.67	0.62	0.74	0.70	0.77	0.69	0.71	0.79	**0.88**	0.87
DRB1*0301	0.66	0.53		0.65	0.67	0.69		0.50	0.72	**0.82**	**0.82**
DRB1*0401	0.67	0.52	0.60	0.69	0.63	0.68	0.66	0.65	0.69	**0.73**	0.72
DRB1*0404	0.72	0.64		0.79	0.66	0.75			0.80	**0.83**	**0.83**
DRB1*0405	0.67	0.51		0.75	0.62	0.69	0.62		0.72	**0.81**	0.80
DRB1*0701	0.69		0.63	0.78	0.58	0.78		0.68	0.83	0.86	**0.87**
DRB1*0802	0.74	0.70		0.77		0.75			0.82	0.79	**0.81**
DRB1*0901	0.62	0.48			0.61	0.66		0.73	0.68	0.68	**0.69**
DRB1*1101	0.73	0.60		0.80	0.70	0.81			0.80	**0.89**	**0.89**
DRB1*1302	0.79	0.54		0.58	0.52	0.69			0.73	**0.78**	**0.78**
DRB1*1501	0.70	0.63		0.72	0.62	0.74	0.64	0.67	0.72	**0.77**	0.76
DRB3*0101	0.59					0.68				0.85	**0.86**
DRB4*0101	0.74	0.61			0.65	0.71			0.74	**0.86**	**0.86**
DRB5*0101	0.70	0.59		0.79	0.73	0.75	0.63		0.79	**0.87**	**0.87**

**Ave**	0.70	0.59	0.62	0.73	0.64	0.73	0.65	0.66	0.76	0.82	0.82

*NN-W-P1 *is the NN-based method including data redundancy step-size rescaling and P1-PSSM encoding and *NN-W *is the NN-based method including data redundancy step-size rescaling. Both methods were evaluated using 10-fold cross-validation. The performance values for the 9 other methods were taken from Wang et al. [18]. For each allele, the best performing NN method is highlighted in bold and the best performing of all methods is underlined.

### The Lin et al. benchmark data set

The predictive performance of the *NN-W-P1 *and *NN-W *methods was next evaluated on the Lin et al. benchmark data set [24]. The result of this benchmark is shown in Table 5. All methods from the Lin benchmark that cover at least six alleles and that were labeled as "best performing" by Lin at al. were included in the benchmark. Also from this benchmark, it is apparent that the *NN-W-P1 *and *NN-W *methods have similar predictive performance and that they outperformed all other methods in the benchmark.

**Table 5:** Predictive performance in terms of the AUC on the Lin benchmark data set.

Method	DRB1*0101	DRB1*0301	DRB1*0401	DRB1*0701	DRB1*1101	DRB1*1501	Ave
*IEDB_SMM*	0.81	0.71	0.79	0.67	0.84	0.67	0.75
*IEDB_SAT*	0.89	0.69	0.75	0.74	0.83	0.66	0.76
*IEDB_Cons*	0.83	0.67	0.72	0.80	0.84	0.66	0.75
*Multipred_SVM*	0.86	0.80	0.65	0.70	0.78	0.62	0.74
*NetMHCII*	0.77	0.69	0.81	0.62	0.78	0.65	0.72
*NetMHCIIpan*	0.84	0.65	0.81	0.83	0.81	0.79	0.79
*Propred*	0.89	0.70	0.75	0.74	0.83	0.66	0.76
*SVMHC*	0.86	0.69	0.75	0.74	0.83	0.66	0.76
*SYF*	0.72	0.65	0.69	0.70	0.59	0.77	0.69

*NN-W-P1*	**0.90**	0.78	**0.84**	0.75	**0.85**	0.79	0.82
*NN-W*	**0.90**	**0.79**	0.80	**0.76**	0.83	**0.82**	0.81

*NN-W-P1 *is the NN-based method including data redundancy step-size rescaling and P1-PSSM encoding and *NN-W *the NN-based method including data redundancy step-size rescaling. All other methods are described in the Lin et al. publication. The AUC was calculated using the following binding affinity threshold values for each of the 6 alleles: DRB1*0101, 0401, 0701, and 1501 threshold = 100 nM, DRB1*0301, 1101, and 1301, threshold = 1000 nM (Lin HH personal communication). The performance values for the 9 methods above the dotted line were taken from Lin et al. [24]. For each allele, the best performing NN method is highlighted in bold and the best performing method is underlined.

### The El-Manzalawy benchmark data set

The recent benchmark by El-Manzalawy and co-workers [25] demonstrated that the predictive performance of a given prediction method depends strongly on the inherent peptide similarity in the training data and that a benchmark ranking of peptide binding prediction algorithms could vary as a function of this similarity. To investigate to what extent the *NN-align *method and its performance were influenced by peptide similarities, we compared the performance of the *NN-W-P1 *and *NN-W *methods to the MHC class II binding prediction methods (*5-spectrum, LA, and CTD*) included in the El-Manzalawy paper on three data sets, each with a different degree of inherent sequence similarity. From the results summarized in Table 6 (details are given in supplementary material [see Additional file 2]) it is clear that the NN-based methods significantly outperformed the three other methods included in the benchmark, independent of the inherent peptide similarity in the training data. Only for one single comparison, *NN-W-P1 *versus *CTD*, the difference was only marginally significant (p < 0.06). The *NN-W *method had the highest predictive performance. The difference to *NN-W-P1 *was, however, only significant for the SRDS2 data set (p < 0.05). This analysis demonstrated that the NN-based methods are robust and perform well also in situations where the peptide similarity is low.

**Table 6:** Predictive performance in terms of the AUC on the IEDB El-Manzalawy benchmark.

	UPDS	SRDS1	SRDS2
*NN-W-P1*	0.863	0.699	0.673
*NN-W*	**0.864**	**0.705**	**0.676**
*CTD*	0.782	0.639	0.634
*LA*	0.802	0.645	**0.606**
*5-spectrum*	0.748	0.429	**0.390**

The methods included are *NN-W-P1 *(the NN-based method including data redundancy step-size rescaling and P1-PSSM encoding), *NN-W *(the NN-based method including data redundancy step-size rescaling), CTD, LA, and 5-spectrum. The performance values for the latter three methods are taken from the El-Manzalawy publication [25]. The benchmark data sets are UPDS: Unique peptides from the IEDB database, SRDS1: Sequence similarity reduced UPDS data excluding peptides sharing 9 mer subsequences, and SRDS2: Sequence similarity reduced SRDS1 data ensuring maximum more than 80% similarity between pairs of peptides. For each allele, the best performing NN-based method is highlighted in bold and the best performing method is underlined.

### The NN-align method

From the large set of benchmark studies, we conclude that the two methods *NN-W-P1 *and *NN-W *share comparable predictive performance. The *NN-W-P1 *method was shown to be more accurate in identifying the peptide core binding register, whereas the *NN-W *was more accurate when predicting the binding affinity. However, none of these differences were consistently statistically significant. The final method, called *NN-align*, was therefore implemented with a switch that allows the user to select whether or not to include the P1-PSSM amino acid preference encoding.

## Discussion

Prediction of which peptides will bind to a given MHC molecule has great implications for the understanding of how a given host will react to a pathogen infection. Here, we have developed an artificial neural network-based method, *NN-align*, for MHC class II peptide binding prediction. The *NN-align *method is inspired by the earlier published *Gibbs sampler *[10], *SMM-align *[20], and *NetMHCIIpan *[23] methods. The method is trained on a large set of quantitative peptide binding data consisting of more than 14,000 peptide affinity values covering 14 human MHC class II DR molecules. Each peptide is encoded to the method in terms of the peptide binding core and information about the length and composition of the residues flanking this core. *NN-align *was trained using a novel training algorithm that allows for correction of bias in the training data due to redundant binding core representation. Inclusion of peptide flanking residues was shown to significantly improve the prediction accuracy. The method was benchmarked on six independent data sets and was shown to consistently and significantly outperform other state-of-the-art MHC class II prediction algorithms, like *TEPITOPE*, *SMM-align*, and *NetMHCIIpan*. In particular, it was demonstrated that the *NN-align *method could provide both accurate quantitative predictions that directly translate into IC50 binding values and correct identification of the peptide-binding core.

In contrast to the *SMM-align *method (and other matrix-based methods), *NN-align *is neural network based and can hence take into account higher order sequence correlations. For MHC class I binding, this has been demonstrated to be of importance for accurate prediction of peptide binding to MHC molecules [29]. Furthermore, *NN-align *incorporates PFR and peptide length directly into the training of the method. This is in contrast to *SMM-align*, where the PFR and peptide length are dealt with in an ad-hoc manner. The allele-specific *NN-align *and pan-specific *NetMHCIIpan *methods are highly similar in their design and use of peptide features. The pan-specific method, however, is one single universal method capable of predicting binding for all HLA-DR alleles of known protein sequence, in contrast to the allele-specific method, *NN-align*, that is trained individually for each allele. As observed for MHC class I methods, it is therefore expected that the allele-specific method will outperform the pan-specific method for alleles where sufficient data is available to accurately characterize the binding motif, as is the case for most alleles in the quantitative data set used here [33].

The *NN-align *algorithm, even though here only applied to the problem of peptide MHC class II binding prediction based on peptide data points, should be generally applicable to find subtle linear sequence motifs in large scale quantitative data sets like peptide chip data for MHC binding, TCR recognition, monoclonal antibodies etc., where the precise location of the receptor peptide interaction is not *a priori *known [34].

## Conclusion

We have developed a method, *NN-align*, for prediction of peptide binding to MHC class II molecules. *NN-align *was trained using a novel training algorithm that allowed for correction of bias in the training data due to redundant binding core representation. The predictive performance of the method was significantly improved by incorporation of information from residues flanking the peptide-binding core. Large scale benchmarking comprising six independent data sets covering 14 human MHC class II alleles demonstrated that *NN-align *outperformed other state-of-the-art MHC class II prediction methods. The method and benchmark data are made publicly available at http://www.cbs.dtu.dk/services/NetMHCII-2.0.

## Authors' contributions

MN developed the *NN-align *method, designed the benchmark, trained the prediction method and did the performance comparison between the different prediction methods, and drafted the manuscript. All authors read and corrected the manuscript.

## Supplementary Material

Additional file 1 **Predictive performance for 14 HLA-DR alleles in the quantitative IEDB benchmark data set**. The performance is estimated using five-fold cross-validation, and the predictive performance is estimated in terms of the Pearson's correlation. *SMM-align *is the *SMM-align *method described by Nielsen et al. [20] re-trained on the quantitative benchmark data set. *NN *is the standard NN-based method, *NN-W *is the NN-based method including data redundancy step-size rescaling, *NN-P1 *is the NN-based method including PSSM-P1 amino acid encoding, *NN-W-P1 *is the NN-based method including step-size rescaling and P1 amino acid encoding. *NN-xPFR *is the *NN-W-P1 *method excluding peptide-flanking residue encoding. For each allele, the best performing NN method is highlighted in bold and the best performing of all methods is underlined. Click here for file

Additional file 2 **Predictive performance in terms of the AUC on the IEDB El-Manzalawy benchmark**. The methods included are *NN-W-P1 *(the NN-based method including data redundancy step-size rescaling and P1-PSSM encoding), *NN-W *(the NN-based method including data redundancy step-size rescaling), CTD, LA, and 5-spectrum. The performance values for the latter three methods are taken from the El-Manzalawy publication [25]. The benchmark data sets are UPDS: Unique peptides from the IEDB database, SRDS1: Sequence similarity reduced UPDS data excluding peptides sharing 9 mer subsequences, and SRDS2: Sequence similarity reduced SRDS1 data ensuring maximum more than 80% sequence similarity between pairs of peptides. For each allele, the best performing of all methods is underlined. Click here for file
