# Supplementary material for: NN-align. An artificial neural network-based alignment algorithm for MHC class II peptide binding prediction
Source: BMC Bioinformatics. 2009 Sep 18;10:296. doi: 10.1186/1471-2105-10-296 (PMC2753847; doi:10.1186/1471-2105-10-296)
Supplement: Additional file 1 — Predictive performance for 14 HLA-DR alleles in the quantitative IEDB benchmark data set. The performance is estimated using five-fold cross-validation, and the predictive performance is estimated in terms of the Pearson's correlation. SMM-align is the SMM-align method described by Nielsen et al. [20] re-trained on the quantitative benchmark data set. NN is the standard NN-based method, NN-W is the NN-based method including data redundancy step-size rescaling, NN-P1 is the NN-based method including PSSM-P1 amino acid encoding, NN-W-P1 is the NN-based method including step-size rescaling and P1 amino acid encoding. NN-xPFR is the NN-W-P1 method excluding peptide-flanking residue encoding. For each allele, the best performing NN method is highlighted in bold and the best performing of all methods is underlined. [file 1471-2105-10-296-S1.DOC]

**Supplementary Table S1. Predictive performance 14 HLA-DR alleles in the quantitative IEDB benchmark data set.**

| **Allele** | **#** | ***SMM-align*** | ***NN*** | ***NN-W*** | ***NN-P1*** | ***NN-W-P1*** | ***NN-xPFR*** |
| --- | --- | --- | --- | --- | --- | --- | --- |
| DRB1*0101 | 5166 | 0.610 | **0.683** | **0.683** | 0.677 | 0.676 | 0.640 |
| DRB1*0301 | 1020 | 0.563 | 0.591 | **0.601** | 0.594 | 0.596 | 0.532 |
| DRB1*0401 | 1024 | 0.496 | 0.533 | **0.536** | 0.525 | 0.525 | 0.520 |
| DRB1*0404 | 663 | 0.579 | 0.621 | **0.628** | 0.616 | 0.616 | 0.610 |
| DRB1*0405 | 630 | 0.560 | 0.530 | **0.548** | 0.545 | 0.547 | 0.542 |
| DRB1*0701 | 853 | 0.618 | **0.680** | 0.675 | 0.668 | 0.668 | 0.627 |
| DRB1*0802 | 420 | 0.555 | 0.600 | 0.587 | 0.604 | **0.610** | 0.558 |
| DRB1*0901 | 530 | 0.360 | 0.286 | 0.280 | 0.280 | **0.30**5 | 0.274 |
| DRB1*1101 | 950 | 0.581 | **0.642** | **0.642** | 0.634 | 0.625 | 0.609 |
| DRB1*1302 | 498 | 0.558 | 0.624 | **0.635** | 0.627 | 0.626 | 0.605 |
| DRB1*1501 | 934 | 0.528 | 0.571 | **0.582** | 0.575 | 0.573 | 0.568 |
| DRB3*0101 | 549 | 0.585 | 0.585 | **0.586** | 0.573 | 0.574 | 0.558 |
| DRB4*0101 | 446 | 0.541 | 0.577 | 0.577 | 0.585 | **0.589** | 0.588 |
| DRB5*0101 | 924 | 0.529 | 0.587 | **0.602** | 0.585 | 0.583 | 0.570 |
| **Ave** |  | 0.547 | 0.579 | 0.583 | 0.578 | 0.580 | 0.557 |

The performance is estimated using five-fold cross-validation, and the predictive performance is estimated in terms of the Pearson’s correlation. SMM-align is the SMM-align method described by Nielsen et al. [20] re-trained on the quantitative benchmark data set. NN is the standard NN-based method, NN-W is the NN-based method including data redundancy step-size rescaling, NN-P1 is the NN-based method including PSSM-P1 amino acid encoding, NN-W-P1 is the NN-based method including step-size rescaling and P1 amino acid encoding. NN-xPFR is the NN-W-P1 method excluding peptide-flanking residue encoding. For each allele, the best performing NN method is highlighted in bold and the best performing of all methods is underlined.
