# Supplementary material for: NN-align. An artificial neural network-based alignment algorithm for MHC class II peptide binding prediction
Source: BMC Bioinformatics. 2009 Sep 18;10:296. doi: 10.1186/1471-2105-10-296 (PMC2753847; doi:10.1186/1471-2105-10-296)
Supplement: Additional file 2 — Predictive performance in terms of the AUC on the IEDB El-Manzalawy benchmark. The methods included are NN-W-P1 (the NN-based method including data redundancy step-size rescaling and P1-PSSM encoding), NN-W (the NN-based method including data redundancy step-size rescaling), CTD, LA, and 5-spectrum. The performance values for the latter three methods are taken from the El-Manzalawy publication [25]. The benchmark data sets are UPDS: Unique peptides from the IEDB database, SRDS1: Sequence similarity reduced UPDS data excluding peptides sharing 9 mer subsequences, and SRDS2: Sequence similarity reduced SRDS1 data ensuring maximum more than 80% sequence similarity between pairs of peptides. For each allele, the best performing of all methods is underlined. [file 1471-2105-10-296-S2.DOC]

**Supplementary Table S2. Predictive performance in terms of AUC on the IEDB El-Manzalawy benchmark.**

| UPDS |  |  |  |  |  |
| --- | --- | --- | --- | --- | --- |
| Allele | *NN-W* | *NN-W-P1* | *SVM* | *LA* | *CDT* |
| DRB1-0101 | 0.831 | **0.832** | 0.806 | 0.767 | 0.770 |
| DRB1-0301 | 0.880 | **0.885** | 0.771 | 0.822 | 0.788 |
| DRB1-0401 | **0.874** | 0.869 | 0.769 | 0.786 | 0.790 |
| DRB1-0404 | 0.873 | **0.877** | 0.639 | 0.763 | 0.821 |
| DRB1-0405 | 0.856 | **0.858** | 0.693 | 0.770 | 0.788 |
| DRB1-0701 | **0.876** | 0.870 | 0.727 | 0.799 | 0.757 |
| DRB1-0802 | **0.882** | 0.874 | 0.703 | 0.896 | 0.793 |
| DRB1-1101 | **0.887** | 0.880 | 0.800 | 0.822 | 0.798 |
| DRB1-1302 | 0.869 | **0.875** | 0.783 | 0.865 | 0.775 |
| DRB1-1501 | **0.848** | 0.842 | 0.794 | 0.781 | 0.779 |
| DRB4-0101 | 0.841 | **0.852** | 0.703 | 0.795 | 0.738 |
| DRB5-0101 | **0.847** | 0.842 | 0.784 | 0.755 | 0.785 |
| Ave | 0.864 | 0.863 | 0.748 | 0.802 | 0.782 |
|  |  |  |  |  |  |
| SRDS1 |  |  |  |  |  |
| Allele | *NN-W* | *NN-W-P1* | *SVM* | *LA* | *CDT* |
| DRB1-0101 | **0.673** | 0.665 | 0.427 | 0.628 | 0.604 |
| DRB1-0301 | 0.715 | **0.724** | 0.460 | 0.668 | 0.605 |
| DRB1-0401 | **0.694** | 0.685 | 0.444 | 0.619 | 0.671 |
| DRB1-0404 | 0.773 | **0.781** | 0.411 | 0.644 | 0.703 |
| DRB1-0405 | **0.689** | 0.670 | 0.331 | 0.557 | 0.654 |
| DRB1-0701 | **0.746** | 0.742 | 0.445 | 0.658 | 0.605 |
| DRB1-0802 | **0.718** | 0.709 | 0.378 | 0.762 | 0.692 |
| DRB1-1101 | **0.714** | 0.710 | 0.454 | 0.668 | 0.698 |
| DRB1-1302 | **0.730** | 0.724 | 0.549 | 0.750 | 0.627 |
| DRB1-1501 | 0.664 | **0.677** | 0.460 | 0.595 | 0.623 |
| DRB4-0101 | **0.680** | 0.670 | 0.444 | 0.611 | 0.547 |
| DRB5-0101 | **0.664** | 0.634 | 0.344 | 0.578 | 0.638 |
| Ave | 0.705 | 0.699 | 0.429 | 0.645 | 0.639 |
|  |  |  |  |  |  |
| SRDS2 |  |  |  |  |  |
| Allele | *NN-W* | *NN-W-P1* | *SVM* | *LA* | *CDT* |
| DRB1-0101 | **0.668** | 0.659 | 0.436 | 0.629 | 0.664 |
| DRB1-0301 | 0.680 | **0.691** | 0.401 | 0.657 | 0.673 |
| DRB1-0401 | **0.653** | 0.645 | 0.371 | 0.592 | 0.662 |
| DRB1-0404 | 0.733 | **0.749** | 0.364 | 0.578 | 0.664 |
| DRB1-0405 | 0.620 | **0.638** | 0.340 | 0.523 | 0.576 |
| DRB1-0701 | 0.719 | **0.725** | 0.389 | 0.611 | 0.609 |
| DRB1-0802 | **0.733** | 0.702 | 0.370 | 0.732 | 0.732 |
| DRB1-1101 | **0.712** | 0.707 | 0.399 | 0.602 | 0.666 |
| DRB1-1302 | **0.690** | 0.679 | 0.483 | 0.681 | 0.594 |
| DRB1-1501 | **0.601** | 0.599 | 0.380 | 0.534 | 0.636 |
| DRB4-0101 | **0.692** | 0.670 | 0.466 | 0.627 | 0.644 |
| DRB5-0101 | 0.606 | **0.610** | 0.278 | 0.503 | 0.492 |
| Ave | 0.676 | 0.673 | 0.390 | 0.606 | 0.634 |

The methods included are NN-W-P1 (the NN-based method including data redundancy step-size rescaling and P1-PSSM encoding), NN-W (the NN-based method including data redundancy step-size rescaling), CTD, LA, and 5-spectrum. The performance values for the latter three methods are taken from the El-Manzalawy publication [25]. The benchmark data sets are UPDS: Unique peptides from the IEDB database, SRDS1: Sequence similarity reduced UPDS data excluding peptides sharing 9mer subsequences, and SRDS2: Sequence similarity reduced SRDS1 data ensuring maximum more than 80% sequence similarity between pairs of peptides. For each allele, the best performing of all methods is underlined.
